# Estimating the shares of the value of branded pharmaceuticals accruing to manufacturers and to patients served by health systems

**DOI:** 10.1002/hec.4393

**Published:** 2021-08-02

**Authors:** Beth Woods, Aimée Fox, Mark Sculpher, Karl Claxton

**Affiliations:** ^1^ Centre for Health Economics University of York York UK

**Keywords:** cost‐effectiveness, pharmaceuticals, value‐based pricing

## Abstract

Previous studies have estimated that patients served by health systems accrue 59‐98% of the value generated by new pharmaceuticals. This has led to questions about whether sufficient returns accrue to manufacturers to incentivize socially optimal levels of R&D. These studies have not, however, fully reflected the health opportunity costs imposed by payments for branded pharmaceuticals. We present a framework for estimating how the value generated by new branded pharmaceuticals is shared. We quantify value in net health effects and account for benefits and health opportunity costs in the patent period and post‐patent period when generic/biosimilar products become available. We apply the framework to 12 National Institute for Health and Care Excellence appraisals and show that realized net health effects range from losses of 160%, to gains of 94%, of the potential net health benefits available. In many cases, even in the long run, the benefits of new medicines are not sufficient to offset the opportunity costs of payments to manufacturers, and approval is expected to reduce population health. This cannot be dynamically efficient as it incentivizes future innovation at prices which will also reduce population health. Further work should consider how to reflect these findings in reimbursement policies.

## 1 INTRODUCTION

A number of studies have addressed the important question of how the value generated by pharmaceuticals is shared between patients served by health systems and drug manufacturers (Camejo et al., 2014; Grabner et al., 2011; Jena & Philipson, 2008; Lindgren & Jonsson, 2012; Moreno & Ray, 2016). These studies estimate that patients served by health systems accrue 59%–98% of the total value generated by new drugs and manufacturers accrue the remaining 2%–41% of value. This has led to questions about whether this return is sufficient to incentivize socially optimal levels of R&D investments; that is whether current policy will lead to dynamic efficiency (Jena & Philipson, 2008).

These studies estimate total value by valuing health gains according to an approval norm (often called a cost‐effectiveness threshold) or an estimate of the consumption value of health (estimated as individuals' willingness to pay for a quality‐adjusted life year [QALY] or value per statistical life) and compare this to payments for the branded product. This approach does not acknowledge the difference between approval norms or estimates of the consumption value of health, and estimates of the likely health opportunity costs of payments for branded products. Empirical evidence now indicates that the health opportunity costs of health care funds are considerably higher than existing approval norms or estimates of consumption value suggest (Claxton et al., 2015; Lomas et al., 2019; Ochalek et al., 2018). The proportion of value accruing to manufacturers is, therefore, likely to be higher than previously estimated.

In this study we set out (1) a general framework for estimating the total value of a new branded drug and how this is shared between patients served by health systems and manufacturers; (2) the evidence required to support an empirical assessment of value and how this is shared and the nature of this evidence in the UK context; and (3) the results of applying this framework to a sample of products approved by the National Institute for Health and Care Excellence (NICE) in England.

## 2 FRAMEWORK FOR ESTIMATING VALUE AND HOW THIS IS SHARED

**FIGURE 1:**
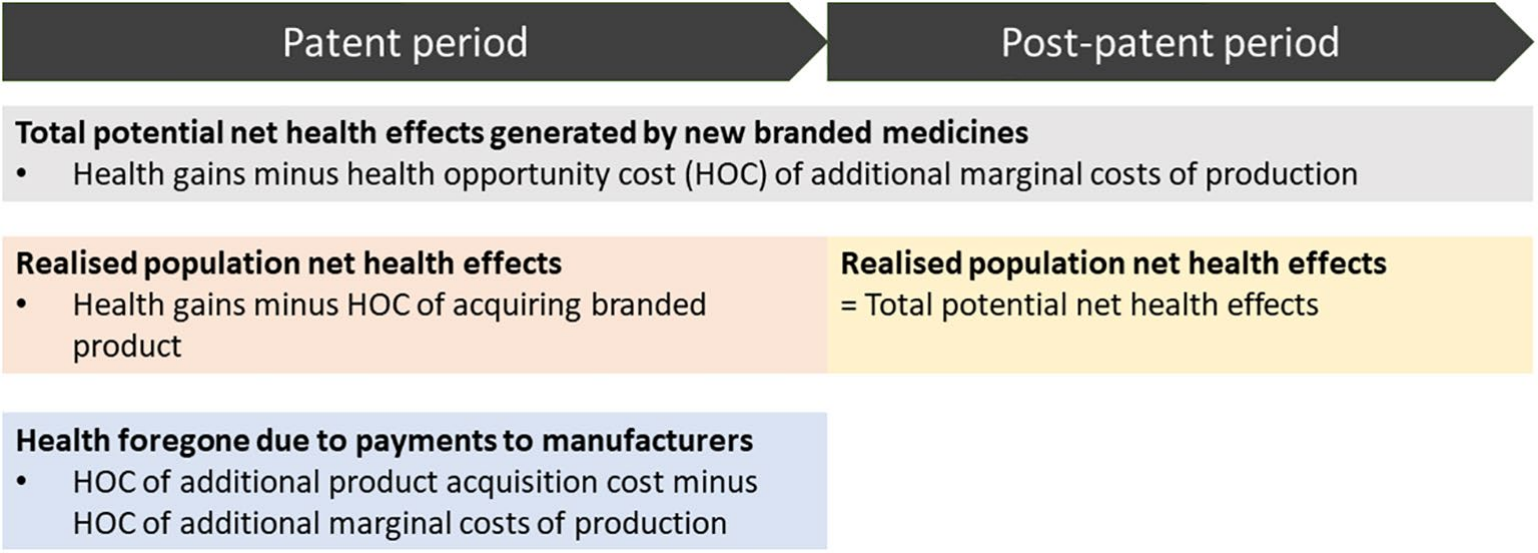
Simplified schematic of value accruing to different parties in health terms. This schematic assumes generic prices are equal to production costs, generic uptake is immediate and complete at loss of patent protection, and no additional costs to the health system are associated with using the product. These assumptions are revisited in the course of this study. HOC, health opportunity cost

Figure 1 shows how the value accruing to each party is calculated. We describe the framework for contexts in which pricing and reimbursement decisions are based on cost‐effectiveness, and technologies are assessed as cost‐effective if their incremental cost‐effectiveness ratio (ICER) is below an approval norm (often referred to as a cost‐effectiveness threshold). The framework can also be applied in contexts where different approaches to pricing and reimbursement are used, as long as evidence relating to cost‐effectiveness is available.

We measure value using net health effects, which account for both the health gains from using a new drug and the health opportunity costs associated with the additional costs of funding it. Costs to the health system can be expressed as health foregone using a measure of health opportunity cost, k. For example, if k is £15,000/QALY this implies that for every £15,000 of health care resources used to fund a treatment, 1 QALY of health is displaced elsewhere in the health system.

### 2.1 Total potential net health effects generated by new branded medicines

The total potential net health effects generated by a new medicine per treated patient is the health gains generated by the product (Δh), compared to the relevant comparator, less the health opportunity costs associated with the additional marginal costs of producing the drug (Δmc), again, compared to the relevant comparator:

(1) $$\begin{array}{l} \Delta h-\frac{\Delta \mathrm{mc}}{k} \end{array}$$

Health benefits and marginal costs accrue over different timeframes depending on the drug and clinical indication. The values presented in Equation (1) reflect total lifetime values discounted to the present. To simplify exposition, in this section we assume there are no other incremental costs to the health care system associated with introducing the new drug. We revisit this assumption in Section 2.4.

The net health effects accrue each time a patient initiates the new treatment. Assuming a constant annual number of patients presenting for treatment (*n*_*t*_), the total potential discounted lifetime net health effects generated by the drug are:

(2) $$\begin{array}{l} \overset{T=\infty }{\underset{t=1}{\sum }}\frac{n_{t}}{{\left(1+r\right)}^{t}}\left(\Delta h-\frac{\Delta \mathrm{mc}}{k}\right) \end{array}$$

where *r* is the overall annual discount rate considered relevant to comparing health interventions accruing benefits and costs over different time frames. As show in Equation (2), net health effects accrue over an infinite time horizon. In practice, usage may decline as new products emerge and products will often eventually become obsolete. However, so long as the value of these new drugs are assessed relative to existing comparators, the contribution of older treatments to population health “lives on” even once they have been replaced by new and better alternatives.

### 2.2 Realized population net health effects

We now consider the net health effects that accrue to patients served by health systems, taking in to account the price paid for branded medicines. At launch, companies face incentives to achieve the highest price that would permit approval. We therefore assume that the incremental drug cost (i.e., the price premium compared to the comparator) is set equal to Δh⋅λ where λ is the approval norm. This approval norm is often described as a cost‐effectiveness threshold. In the patent period, patients served by the health system accrue the health gains minus the health opportunity costs of the additional payments for the branded product:

(3) $$\begin{array}{l} \Delta h-\frac{\Delta h\cdot \lambda }{k} \end{array}$$

This is positive if the approval norm (*λ*) is less than the measure of health opportunity cost(k), and negative if the approval norm exceeds the measure of health opportunity cost. We use the term “patent period” throughout the study, we intend this to represent the period over which a broader suite of intellectual property rights protects new drugs from generic/biosimilar competition.

For the time being, we assume that once the drug comes off patent it is sold at the marginal cost of production by the original manufacturer or competitors selling generics/biosimilars. Post‐patent patients served by the health system accrue all available net health gains (as shown in Equation 1). This will be positive as long as the drug delivers net health gains at generic/biosimilar prices, that is, as long as the ICER of the drug at generic/biosimilar prices is below the measure of health opportunity cost.

The realized population net health effects including both patent and post‐patent periods are:

(4) $$\begin{array}{l} \overset{T=t_{p}}{\underset{t=1}{\sum }}\frac{n_{t}}{{\left(1+r\right)}^{t}}\left(\Delta h-\frac{\Delta h\cdot \lambda }{k}\right)+\overset{T=\infty }{\underset{t=t_{p}+1}{\sum }}\frac{n_{t}}{{\left(1+r\right)}^{t}}\left(\Delta h-\frac{\Delta \mathrm{mc}}{k}\right)\; \end{array}$$

where $t_{p}$ denotes the duration of the patent.

If this drug is supplanted by another new medicine (which we call here “*new 2*”), the value described in Equation (4) for “*new 1*” will continue to accrue to patients served by health systems as long as *new 2* is priced relative to the generic version of *new 1* once *new 1*'s patent expires.

### 2.3 Health foregone due to payments to manufacturers

We convert value accruing to manufacturers, which is most naturally considered in monetary terms, to health foregone by patients to allow comparisons of value accruing to each party using a common numeraire. The health foregone due to payments to manufacturers is calculated as the opportunity cost of the incremental price premium paid for the drug minus the health opportunity costs imposed by the incremental marginal cost of production:

(5) $$\begin{array}{l} \frac{\Delta h\cdot \lambda }{k}-\frac{\Delta \mathrm{mc}}{k} \end{array}$$

This accrues throughout the patent period:

(6) $$\begin{array}{l} \overset{T=t_{p}}{\underset{t=1}{\sum }}\frac{n_{t}}{{\left(1+r\right)}^{t}}\left(\frac{\Delta h\cdot \lambda }{k}-\frac{\Delta \mathrm{mc}}{k}\right)\; \end{array}$$

If, within its patent period, this drug is supplanted by another new medicine (*new 2*) the value described in Equation (6) for *new 1* will accrue to the *new 2* manufacturer. Equation (6) therefore represents the overall value accruing to all originator brand manufacturers rather than necessarily the value accruing to the manufacturer of the appraised drug. How this value should be split between originator manufacturers is beyond the scope of this study.

### 2.4 Incorporating additional health system costs

Appendix A shows how the framework changes when additional health system costs or savings are included. Additional health‐care costs reduce the price premium to the manufacturer during the patent period and reduce population health gains in the post‐patent period. Cost savings increase payments to the manufacturer during the patent period and increase population health gains in the post‐patent period.

### 2.5 Numeric example

We now illustrate this framework using a simple numeric example with data as shown in Table 1. This numeric example is based on the UK context, however the numbers used are intended to simplify the numeric illustration and should not be taken as representing parameter estimates. For example, 10 years is shorter than the average period of patent protection afforded to new medicines which is estimated to be closer to 13 years (Copenhagen Economics, 2018). We discuss data available to inform empirical estimates of value and value shares in Section 3.

**TABLE 1:** Parameters used in numeric example

Parameter	Description	Value
Δh	Incremental health gain (QALYs)	0.50
k	Measure of health opportunity cost (expenditure to gain one QALY)	£15,000
$t_{P}$	Patent duration in years	10
Δmc	Incremental marginal cost	£0
$n_{t}$	Population treated in each year	1000
r	Annual discount rate for costs and health outcomes	3.5%

Abbreviation: QALY, quality‐adjusted life year.

We use a time horizon of 100 years throughout this study as beyond this point discounting means that any additional value generated by the drug will be minimal.

**FIGURE 2:**
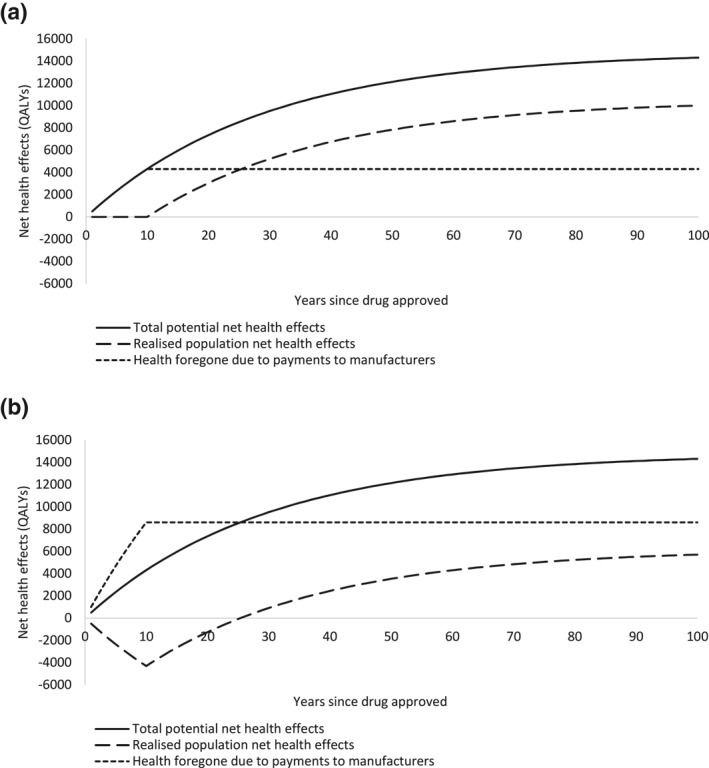
Net health effects accruing to different parties over time for (a) 15,000/quality‐adjusted life year (QALY) approval norm, and (b) 30,000/QALY approval norm for numeric example

Table 2 shows total potential net health effects, realized population net health effects and health foregone due to payments to manufacturers, when the approval norm reflects the measure of health opportunity cost (λ=k). The manufacturer receives a price premium relative to the comparator of £7500 per treated patient (0.5 QALYs × £15,000/QALY). All available health gains (0.5 QALYs per patient treated) accrue as payments to the manufacturer in the patent period as the price is set such that the health opportunity costs of payments to the manufacturer just offset the health gains from using the product. There is no population health gain in the patent period and the population gains the full health benefits of the drug (0.5 QALYs per patient treated) in the post‐patent period. Population health gain is 10,008 QALYs representing 70% of the total potential net health gains, and the health opportunity cost of payments to the manufacturer is 4304 QALYs (30% of the total). The cumulative value accruing to each party is shown in Figure 2a. For simplicity, these graphs show discounted lifetime net health effects for patients who initiate treatment in a given year as accruing within that year, in reality these benefits will be spread over a longer time horizon.

**TABLE 2:** Results of numeric example with approval norm set equal to measure of health opportunity cost (*λ* = *k* = £15,000/QALY)

Metric	Patent period	Post‐patent	Total
Per patient values			
Total potential net health effects	0.50	0.50	
Realized population net health effects	0.00	0.50	
Health foregone due to payments to manufacturer	0.50	0.00	
Total values accrued over time (undiscounted)			
Total potential net health effects	5000	45,000	50,000
Realized population net health effects	0	45,000	45,000
Health foregone due to payments to manufacturer	5000	0	5000
Total values accrued over time (discounted)			
Total potential net health effects	4304	10,008	14,312
Realized population net health effects	0	10,008	10,008
Health foregone due to payments to manufacturer	4304	0	4304

*Note:* All values are net health effects in QALYs.

Abbreviations: *k*, measure of health opportunity cost; *λ*, approval norm; QALY, quality‐adjusted life year.

Table 3 shows what happens when the approval norm is £30,000/QALY. The total potential net health effects are unchanged; however, the higher approval norm changes how value is shared. The manufacturer receives a price premium of £15,000 per treated patient (0.5 QALYs × £30,000/QALY). This payment to the manufacturer imposes an opportunity cost of 1 QALY per patient treated which exceeds the health gains generated from using the product (0.5 QALYs per patient treated). Therefore, during the patent period there is a loss in population health of 0.5 QALYs per patient treated. In the post‐patent period, the health system accrues all health gains as in Table 2. Population health gain is 5704 QALYs which represents 40% of the total potential net health gains, and the health opportunity cost of payments to the manufacturer is 8608 QALYs (60% of the total). The cumulative value accruing to each party is shown in Figure 2b. This shows that it takes until year 26 for the population health gains accrued during the post‐patent period to offset the health losses in the patent period.

**TABLE 3:** Results of numeric example with approval norm set higher than measure of health opportunity cost (*λ* = 30,000/QALY, *k* = £15,000/QALY)

Metric	Patent period	Post‐patent	Total
Per patient values			
Total potential net health effects	0.50	0.50	
Realized population net health effects	−0.50	0.50	
Health foregone due to payments to manufacturer	1.00	0.00	
Total values accrued over time (undiscounted)			
Total potential net health effects	5000	45,000	50,000
Realized population net health effects	−5000	45,000	40,000
Health foregone due to payments to manufacturer	10,000	0	10,000
Total values accrued over time (discounted)			
Total potential net health effects	4304	10,008	14,312
Realized population net health effects	−4304	10,008	5704
Health foregone due to payments to manufacturer	8608	0	8608

*Note:* All values are net health effects in QALYs.

Abbreviations: *k*, measure of health opportunity cost; *λ*, approval norm; QALY, quality‐adjusted life year.

**FIGURE 3:**
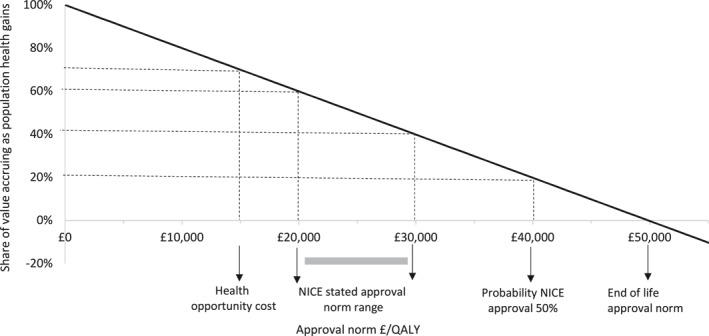
Share of value accruing as population health gains at a range of approval norms relevant to National Institute for Health and Care Excellence (NICE) decision‐making. Results reflect numeric example in Tables 1, 2, 3. QALY, quality‐adjusted life year

Figure 3 shows the proportion of total potential value accruing as population health gains, at a range of approval norms considered relevant to the UK: £15,000/QALY represents a reasonable estimate of the opportunity cost of NHS funds,^1^ £20,000–30,000/QALY is NICE's stated approval norm range (National Institute for Health and Care Excellence, 2013) with higher values within this range reserved for appraisals with particular features, £40,000/QALY reflects an empirical estimate of the ICER above which the probability of rejection by NICE committees exceeds 50% (Dakin et al., 2015), and £50,000/QALY is the approval norm thought to be in operation for products that meet NICE's criteria for life‐extending end‐of‐life (EoL) treatments (Griffiths, 2016). In the numeric example, once the approval norm exceeds £50,000/QALY, the manufacturer accrues more than 100% of the total value and approval of the product reduces population health. This occurs as the health opportunity costs of payments for the branded product during the patent period are not offset by net health gains in the post‐patent period.

### 2.6 Factors determining shares of value

We also explored how a range of factors affect the value accruing to each party. Longer patent durations and other factors that delay availability of generic/biosimilar products or reduce their market penetrance, reduce the share of value accruing as population health gains. The epidemiology of the patient population can change how value is shared by altering the distribution of usage between the patent and post‐patent periods. In clinical indications where incidence increases over time, this increases utilization post‐patent, increasing the share of value accruing as population health gains. An example of this would be antibiotics reserved for the treatment of emerging drug‐resistant infections. A similar effect occurs if prescribing increases once generic/biosimilar versions of the product become available.^2^ If the prevalent population eligible for treatment is relatively large then this focuses usage within the patent period, reducing the share of value accruing as population health gains. For example, a large number of people living with hepatitis C recently became eligible for a series of new drug treatments, whereas long‐term incidence of hepatitis C is expected to be relatively low (NHS, 2019a).

The numeric example above assumes patients initiated on treatment within the patent period will complete their treatment within the patent period. This is appropriate for one‐off treatments and treatments with a relatively short duration. For longer duration treatments, some patients initiated on branded medicines within the patent period may have the latter parts of their treatment course fulfilled by generics/biosimilars. Longer treatment courses therefore increase the share of value accruing as population health gains.^3^

In addition to payments for the branded product, other health system costs also affect how value is shared. Two types of costs are relevant here. The first are additional production costs. For example, although costs of biosimilars are reducing, these drugs remain much costlier than small‐molecule generic drugs due to additional costs associated with development, and production (see Section 3 for further detail). The second relate to the impact of using the new drug on use of other NHS resources, for example, due to treatment administration, required infrastructure, monitoring, or disease management. These can be significant as shown in the recent appraisals of CAR‐T therapies (National Institute for Health and Care Excellence, 2018). We consider how value shares differ for two illustrative drugs that offer the same total potential net health gains but differ in production or NHS costs as shown in Table 4. The first drug delivers higher health gains which are partially offset by additional production or NHS costs. The second drug delivers lower health gains but these are complemented by cost savings which produce health gains elsewhere in the health system. Despite both drugs delivering the same total potential net health gains, the realized population net health gains are lower for the first drug (3982 QALYs, 28% of the total) than the second (7426 QALYs, 52% of the total). When the approval norm exceeds the measure of health opportunity cost, health gains generated directly by the new product are valued more highly than health gains generated via cost savings by a factor of λ/k.

**TABLE 4:** Implications of production and health care costs

Metric	Additional costs scenario	Cost savings scenario
Effects of treatment per patient		
Health gains, QALYs	0.70	0.30
Production or health system costs, £	3000	−3000
Health implications of production/health system costs or savings, QALYs	−0.20	0.20
Net health effects per treated patient, patent period		
Total potential net health effects, QALYs	0.50	0.50
Realized population net health effects, QALYs	−0.70	−0.30
Health foregone due to payments to manufacturer, QALYs	1.20	0.80
Net health effects per treated patient, post‐patent period		
Total potential net health effects, QALYs	0.50	0.50
Realized population net health effects, QALYs	0.50	0.50
Health foregone due to payments to manufacturer, QALYs	0.00	0.00

*Note:* Results for numeric example (*λ* = 30,000/QALY, *k* = £15,000/QALY).

Abbreviations: *k*, measure of health opportunity cost; *λ*, approval norm; QALY, quality‐adjusted life year.

The shares of value are not sensitive to the number of recipient patients or the magnitude of QALY gain^4^ as these rescale the value accruing to the health system and manufacturer proportionately.

Readers can explore the sensitivity of the numeric example to different scenarios and parameter inputs using the Microsoft Excel model provided in the supplementary material.

We now consider how estimates of value and value shares can be produced using data available for the UK setting.

## 3 EVIDENCE REQUIRED TO SUPPORT AN EMPIRICAL ASSESSMENT OF VALUE

The framework outlined above indicates that, in addition to information relating to standard determinants of a product's cost‐effectiveness and a measure of health opportunity cost, assessing the total value of a product, and how this is shared, requires information relating to the timing of availability of generic and biosimilar products, their uptake and pricing. The evidence available to inform these assessments for a series of case study products approved by NICE is described in the following sections.

### 3.1 Evidence for the UK context

#### 3.1.1 Data from NICE appraisals

We apply the framework to 12 NICE technology appraisals. These appraisals were identified from recent work by the NICE Decision Support Unit (DSU) assessing the impact of the EQ‐5D‐5L on cost‐effectiveness estimates (Pennington et al., 2018, 2019). We used case studies from this study as it provided estimates of the ICERs and incremental QALYs corresponding to the final committee decision, and used examples considered broadly representative of the NICE Technology Appraisal program. Importantly, the provision of ICERs and incremental QALYs corresponding to the final committee decision allowed our analysis to fully reflect any confidential discounts that had been agreed as part of the NICE commercial patient access schemes (PAS). The appraisals reflect a wide range of disease areas, biologic and small‐molecule drugs, drugs with and without PAS agreements, approved with and without EoL criteria applying, and represent a mixture of primary and secondary NICE‐approved indications. The estimates are specific to the use of each drug in the indication under appraisal, and the overall value of the drug aggregated across indications will differ. Data required for the analysis were extracted as documented in Appendix B and are shown in Table 5.

**TABLE 5:** Characteristics of included NICE appraisal case studies (ordered by ICER)

TA #	Product (FAD year)	Disease (location of prescribing, whether drug is addition or substitute^a^)	Biologic	PAS	EoL	Multiple indications assessed by NICE?	Approval ICER (£/QALY)	Period on‐patent usage (years)	Incr. costs (£)	Incr. QALYs	Incr. NHS costs (£)	Average treatment duration (months)	Number patients receiving drug	
													Years 1–5^b^	Year 6+
325	Nalmefene (2014)	Alcohol dependence (SC/PC, addition)	No	No	No	No	£1110	11	£76	0.068	−£546	10.0	23,537	35,306
367	Vortioxetine (2015)	Major depressive episodes (SC/PC, substitute)	No	No	No	No	£2970	11	£49	0.017	£0	4.3	50,655	85,961
335	Rivaroxaban (2015)	Acute coronary syndrome (SC/PC, addition)	No	No	No	5th of 5	£5622	7	£675	0.120	£6	12.0	9642	11,505
228^c^	Thalidomide (2010)	Multiple myeloma (SC, addition)	No	No	No	No	£9174	8	£11,192	1.220	£0	11.0	1885	2030
392	Adalimumab (2016)	Hidradenitis suppurativa (SC, addition)	Yes	Yes	No	9th of 11	£19,328	3	£18,168	0.940	−£16,414	23.9	287	0
352	Vedolizumab (2015)	Crohn's disease (SC, addition)	Yes	Yes	No	2nd of 2	£21,620	12	£3892	0.180	−£855	7.4	1175	317
377	Enzalutamide (2015)	Prostate cancer (pre‐chemotherapy) (SC, addition)	No	Yes	No	2nd of 2	£32,985	12	£16,493	0.500	−£11,628	17.7	4195	4724
428	Pembrolizumab (2016)	Non‐small cell lung cancer (SC, substitute)	Yes	Yes	Yes	3rd of 3	£44,490	12	£26,961	0.606	£2568	7.8	1540	1800
391^d^	Cabazitaxel (2016)	Prostate cancer (SC, substitute)	No	Yes	Yes	No	£45,159	11	£10,703	0.237	£0	4.2	370	370
316^e^	Enzalutamide (2014)	Prostate cancer (post‐chemotherapy) (SC, addition)	No	Yes	Yes	1st of 2	£45,626	13	£11,863	0.260	£586	8.5	335	226
357	Pembrolizumab (2015)	Melanoma (SC, addition)	Yes	Yes	Yes	1st of 3	£46,662	13	£41,529	0.890	£1576	9.1	202	226
381	Olaparib (2015)	Ovarian, fallopian tube and peritoneal cancer (SC, addition)	No	Yes	Yes	No	£46,973	12	£37,578	0.800	£2077	20.6	204	251

*Note:* EoL, end of life; FAD, final appraisal determination; ICER, incremental cost‐effectiveness ratio; NICE, National Institute for Health and Care Excellence; PAS, patient access scheme; PC, primary care; QALYs, quality‐adjusted life years; SC, secondary care; TA #, technology appraisal number.

^a^ Variable shows whether new treatment is used in addition to standard of care or is used to substitute for an existing drug.

^b^ Average over years 1–5 presented for brevity, actual annual year 1–5 data used in model.

^c^ This appraisal also recommended bortezomib in patients considered unsuitable for thalidomide. This subgroup is not included here due to a lack of suitable data (see Appendix B).

^d^ We focus on the subgroup of patients who are not eligible for treatment with abiraterone or enzalutamide. Cabazitaxel was also recommended within this appraisal for patients who could receive abiraterone or enzalutamide; however, there was insufficient data to analyze this subgroup (see Appendix B).

^e^ This case study focuses on the subgroup of patients who had received two prior cytotoxic regimens. Enzalutamide was also recommended within this appraisal for patients who had received 1 prior cytotoxic regimen, however there was insufficient data to analyze this subgroup (see Appendix B).

#### 3.1.2 Measure of opportunity cost

We use £15,000/QALY as our measure of the opportunity cost of NHS health care funds. This reflects the most recent empirical evidence on the marginal productivity of the English NHS (Lomas et al., 2019). £15,000/QALY is also routinely used as the measure of health opportunity cost in the Department of Health and Social Care's impact assessments (Department of Health, 2017; Department of Health and Social Care, 2018a, 2018b).

#### 3.1.3 Data relating to the generics and biosimilars market in the UK

Data were also sought on the period of patent protection for each product, and the availability, use and pricing of generic and biosimilar products in the UK. These data are summarized in Table 6. As noted by previous authors, there is an absence of publicly available data on a number of these quantities (Copenhagen Economics, 2018; Ferraro et al., 2018; GaBI Online, 2011). In particular, expected dates for loss of patent protection are not publicly reported for individual drugs. There were also limited relevant data on uptake of generic/biosimilar products and their pricing, so these quantities had to be estimated using additional sub‐analyses and, in some instances, assumptions informed by discussions with individuals with experience of the UK medicines market. Prices for biosimilars reflect publicly available tender prices for adalimumab and infliximab. These tenders achieved significant discounts compared to the corresponding biologic prices and previous prices for biosimilars. Nonetheless, the monthly cost of biosimilars is estimated to exceed the monthly cost of small‐molecule generics by approximately 500%.

**TABLE 6:** Data describing generics and biosimilar availability, usage and acquisition costs in the UK NHS

Parameter	Data sought	Data available
Patent duration	Duration of patent protection from marketing authorization for each appraised drug	Average time from marketing authorization to loss of the last type of patent protection^a^ across Europe for 558 biologic and small‐molecule products with a marketing authorization in the period 1996–2016 (Copenhagen Economics, 2018). Estimated average duration of protection of 13 years.
Delays to market access	Time from market authorization to NHS access for each appraised drug	Time from market authorization to NICE recommendation, or approval via the CDF if earlier, obtained from NICE appraisal documentation and CDF records (NHS, 2020). See Appendix B for data for each appraisal.
Time from loss of exclusivity to entry of generic/biosimilar products	Time to event analysis for different types of products	Time from loss of exclusivity^b^ to first generic medicine being available for 128 top‐selling small molecule drugs prescribed in the community that lost exclusivity in 2000–2007 (European Commission, 2009). Assumed to apply to small‐molecule drugs prescribed in hospitals. See Appendix C for time‐to‐event analysis.
		*De novo* analysis of dates of biologic patent expiry and biosimilar availability using prescribing outlook data (UK Medicines Information, 2012, 2013, 2014, 2015, 2016) for 34 biologic drugs. See Appendix C for further detail and for time‐to‐event analysis.
Uptake of generic/biosimilar products once available	Market share of branded versus generic/biosimilar products following availability of generic/biosimilar for different types of products	No data available for small‐molecule drugs used in hospitals. Assumed 100% uptake given tendering processes in place and centralized dispensing.
		Biologic/biosimilar tendering process (NHS, 2017) assumed to result in similar prices for originator and biosimilar, which is equivalent to assuming 100% uptake for biosimilar.
Pricing of generic/biosimilar products	Price of different types of generic/biosimilar products according to relevant product characteristics	No data available for small‐molecule products used in hospitals. *De novo* analysis of the current generic prices of 16 products appraised by NICE in the period 2000–2004. Monthly costs estimated using regimen data from the appraisals and generic product costs from eMIT (Department of Health and Social Care, 2017) for 2017–2018. Mean monthly cost estimated as £63 (£118 for vials; £30 for tablets). See Appendix D for further details.
		Biologic/biosimilar cost £329 per month based on publicly available tendering documents (National Institute for Health and Care Excellence, 2008, 2010a, 2010b, 2016a, 2016b; NHS, 2019b). See Appendix D for further details.
		Monthly costs are multiplied by the duration of treatment (see Table 5)^c^.

Abbreviations: CDF, cancer drugs fund; NICE, National Institute for Health and Care Excellence.

^a^ This includes the original patent and secondary patents, supplementary protection certificate (SPC), data protection and market protection (including extensions for new therapeutic indications), and further protections for orphan product designations and pediatric investigation plans.

^b^ This includes protection through patents including extensions via an SPC and data exclusivity.

^c^ Where a small‐molecule drug was replacing an existing small molecule drug, the incremental cost of production was assumed to be zero. Where a biologic replaces a small molecule drug, the incremental marginal cost is calculated as the difference between the small molecule and biologic cost. This ignores potential differences in duration of treatment between the existing therapy and comparator but should provide a reasonable approximation.

We assume that generic (and biosimilar) prices reflect underlying marginal costs of production and that, by implication, policy makers and competition authorities have provided an appropriate environment for generic competition. We return to this assumption in the discussion.

The spreadsheet populated with these data is available as a Microsoft Excel model in the supplementary material. This allows users to generate estimates of value and value shares for other products.

## 4 ESTIMATES OF TOTAL NET HEALTH GAINS AND HOW THESE ARE SHARED FOR NICE‐APPROVED PRODUCTS

Estimates of the total potential net health gains for each appraisal, and how these are shared are shown in Table 7. For the four appraisals where the final appraisal determination (FAD) ICER was substantively lower than the measure of opportunity cost (nalmefene, vortioxetine, rivaroxaban and thalidomide), realized population net health effects are 81%–94% of the total potential net health effects. Adalimumab, vedolizumab and enzalutamide initiated in patients who had not received prior chemotherapy had FAD ICERs close to the stated NICE approval norm range of £20,000–30,000/QALY. For enzalutamide initiated in patients who had not received prior chemotherapy, realized population net health gains represent 21% of the total potential net health benefits. For adalimumab and vedolizumab the manufacturer accrues more than 100% of the total potential net health gains and population health is reduced by these approvals. This reflects the approval norm used, the slow emergence of biosimilar drugs and their relatively high cost, and the expected prescribing pattern of these drugs in the appraised indications. Hidradenitis Suppurativa and Crohn's disease are both chronic diseases and use of the drugs is expected to be relatively high in the short term due to the large pool of prevalent cases. The remaining five drugs were considered to meet the EoL criteria by NICE committees and were approved at FAD ICERs of £44,000–47,000/QALY. For all of these appraisals the manufacturer accrues more than 100% of the total potential net health gains. Losses in population health in these appraisals ranged from 18% to 160% of total potential net health gains, and depended on features of the appraisals including whether the drug was a small molecule or biologic product. Across the products considered, the health system accrues 51% of the total potential net health gains.

**TABLE 7:** Value and value share estimates for NICE approved products

Product	FAD ICER (£/QALY)	Total potential net health effects (net QALYs)	Realized population net health effects (net QALYs)	Health foregone due to payments to manufacturers (net QALYs)	Share of value accruing as population health gains (%)	Share of value accruing to manufacturer (%)
Nalmefene	1110	80,460	73,188	7271	91	9
Vortioxetine	2970	37,758	35,165	2593	93	7
Rivaroxaban	5622	30,605	28,774	1831	94	6
Thalidomide	9174	68,772	55,693	13,079	81	19
Adalimumab	19,328	2038	−292	2330	−14	114
Vedolizumab	21,620	991	−432	1423	−44	144
Enzalutamide (pre‐chemotherapy)	32,985	124,410	26,176	98,234	21	79
Pembrolizumab (NSCLC)	44,490	16,360	−26,126	42,486	−160	260
Cabazitaxel	45,159	2510	−456	2966	−18	118
Enzalutamide (post‐chemotherapy)	45,626	1521	−1036	2557	−68	168
Pembrolizumab (melanoma)	46,662	3717	−5312	9029	−143	243
Olaparib	46,973	4317	−2078	6394	−48	148
Total	‐	373,458	183,265	190,193	49	51

*Note:* FAD, final appraisal determination; ICER, incremental cost‐effectiveness ratio; NICE, National Institute for Health and Care Excellence; NSCLC, non‐small‐cell lung cancer; QALY, quality‐adjusted life year.

Three of the products (nalmefene, vortioxetine, and rivaroxaban) may be prescribed in primary care. Results for an analysis assuming primary care prescribing were similar to that shown in Table 7 and are shown in Appendix E.

**FIGURE 4:**
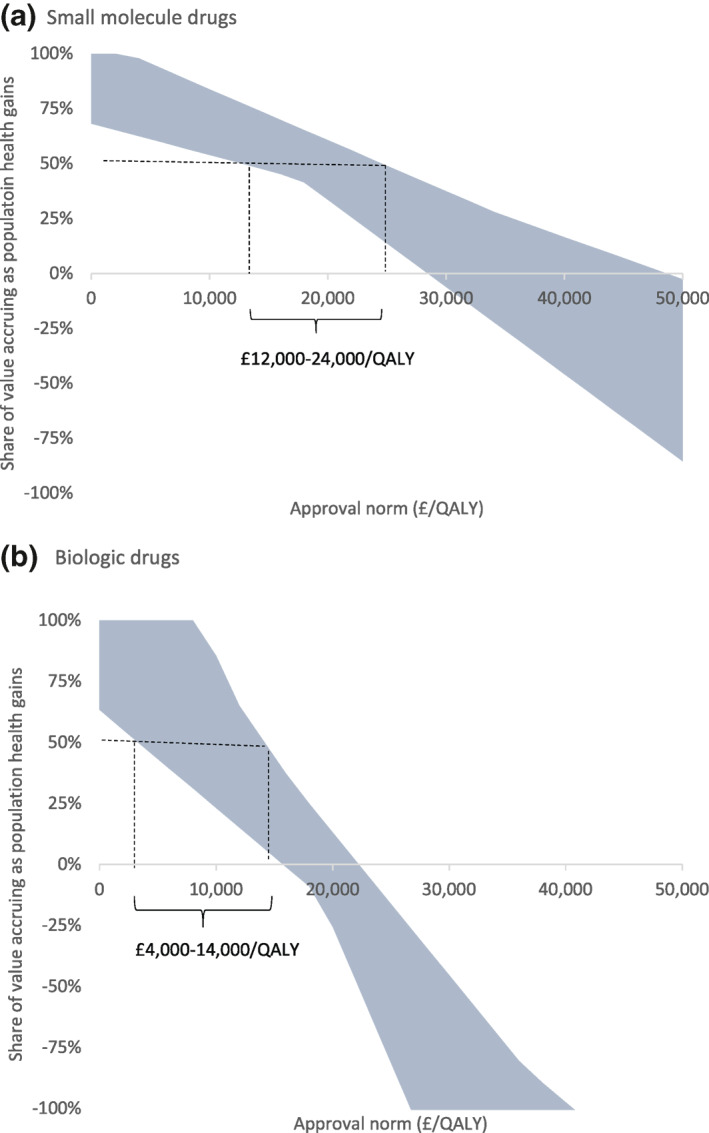
Range of shares of value across (a) small molecule and (b) biologic drugs for different approval norms. The shaded area shows the range of shares of value across the 12 products at different approval norms. The dashed lines indicate the range of approval norms required to deliver a 50% share of value for drugs within each category

We examined how shares of value change if the approval norm is changed, for the small‐molecule and biologic case study appraisals (Figure 4). At any chosen approval norm, there is wide variation in the share of total potential net health effects that accrue as population health effects. If a policy maker intended to achieve a specific share of value, they would need to use different approval norms across products. For example, to achieve a share of 50% would imply an approval norm of £12,000–24,000/QALY for the small‐molecule products examined, and £4000–14,000/QALY for the biologic products examined. Of course, these drugs represent only a sample of those appraised by NICE within each category and the true ranges across all products will be wider.

Decision makers may have other policy tools at their disposal which could improve the share of value accruing to the populations they serve. For example, they may be able to improve access to, and use of, generic and biosimilar products, as well as the price paid for these products. There is some sign that this is already happening for biologics as tendering processes (NHS, 2017) and increased competition improve prices, and policy initiatives and greater acceptance of biosimilar medicines by clinicians improve uptake (NHS Business Services Authority, 2019). We examined the impact of ensuring immediate availability and full uptake of generic/biosimilar products at patent expiry alongside a price reduction of 25% from current levels. Full results of this analysis are shown in Appendix F. These changes result in vedolizumab and adalimumab delivering positive value to the NHS. With these changes, the same shares of value could be achieved at higher approval norms. For example, to achieve a share of 50% would imply an approval norm of £18,000–34,000/QALY for the small‐molecule products and £8000‐20,000/QALY for the biologic products examined.

## 5 DISCUSSION

This work provides a framework for assessing the total potential net health effects of new branded medicines and how these are shared between manufacturers and patients served by health systems. By providing this general framework and accompanying Excel spreadsheets, this framework can be applied to assess value shares internationally.

Our analysis of UK NICE approvals indicates that the share of value accruing to manufacturers is much higher, and the share accruing to patients served by health systems, much lower, than previously estimated (Camejo et al., 2014; Grabner et al., 2011; Jena & Philipson, 2008; Lindgren & Jonsson, 2012; Moreno & Ray, 2016). This work differs from previous studies in that it recognizes that approval norms used as the basis for pricing and reimbursement decisions differ from measures of opportunity cost, and that value continues to accrue even when a product is obsolete as new products build on health gains from existing technologies. For many approvals (seven of the 12 case studies considered here), we found that the manufacturer accrues more than 100% of the total potential net health gains generated by the drug. These approvals therefore lead to reductions in overall population health even in the long run.

A range of factors above and beyond health effects may be considered important when assessing the value of a new medicine. For example, NICE gives extra weight to QALYs generated at the EoL, and the Institute's current methods consultation is considering a range of potential “modifiers” which may influence approval decisions. A number of commentators have emphasized that if these additional factors are used to re‐weight benefits in patients who receive the new drug, then logically the health losses associated with devoting resources to new medicines should be re‐weighted in the same way (Paulden & McCabe, 2021; Sculpher et al., 2017). Incorporation of additional “benefits” can therefore result in medicines appearing both more or less valuable than an analysis focused solely on health effects (Love‐Koh et al., 2019). There is a lack of clarity about whether such additional considerations would be viewed by society as outweighing the substantial health losses associated with many of the approvals.

Our empirical estimates relate to the UK context; however, there is growing evidence across countries that approval norms used to inform drug reimbursement decisions exceed measures of the opportunity costs of health care expenditure (Claxton et al., 2015; Lomas et al., 2019; Ochalek et al., 2018). It may, therefore, be reasonable to expect similar patterns of value sharing in other countries.

Consistent with how economic analysis has tended to inform health‐care decision making, we focus on improvements in population health as the primary objective of health systems (Drummond et al., 2015). Some previous studies have aggregated the benefits of new pharmaceuticals across health system and manufacturers, to assess overall welfare implications (Jena & Philipson, 2008). This is challenging as payments to manufacturers do not equate to producer surplus (due to substantial product development costs) and decision‐making bodies have been unwilling to identify a social welfare function specifying how we might appropriately trade‐off producer surplus with population health.

Notwithstanding these considerations, it is possible to consider the total potential net health effects, and realized population net health effects in terms of consumption value or individuals' willingness to pay (*v*), and to compare these to payments to manufacturers. As total potential net health effects and realized population net health effects are revalued in the same way, this does not change the share of value accruing as population health gains, and therefore our primary conclusions. Importantly, negative net health effects will remain negative regardless of how they are valued. The share of value accruing to manufacturers is reduced to 3%–130% if the consumption value of health is £30,000/QALY and to 1%–65% if it is £60,000/QALY (see Appendix G). This reflects that payments to manufacturers could have generated consumption value at a higher rate (by a factor of *v*/*k*) if retained in the health system and used to generate health.

### 5.1 Additional considerations

The estimates presented are predicated on generic/biosimilar prices eventually being realized. This may happen directly as patients use the generic/biosimilar product, or indirectly, by future products pricing relative to the generic/biosimilar product. This work emphasizes that if a generic/biosimilar version of a comparator is available, we should always price relative to the generic/biosimilar. For some appraisals, comparators remain within patent. In this context it is appropriate to price relative to the branded comparator and then adjust pricing once generic/biosimilar versions of the comparator become available. As long as this re‐pricing occurs, the net health gains associated with the branded comparator will materialize even if it is replaced in clinical practice by the new drug. If re‐pricing does not occur this transfers cost savings that should have accrued to the NHS to the manufacturer of the new product, and reduces population health. This re‐pricing is not, to our knowledge, standard practice in the UK. When appraising medicines with branded comparators, NICE and other reimbursement bodies could usefully pre‐specify the price adjustment required when the generic/biosimilar version of the comparator becomes available. Compared to the status quo, this would be expected to disincentivize R&D efforts in clinical indications where existing branded therapies are being used, and increase incentives to invest in areas where older generic/biosimilar products are being used.

We assumed that prices of generics/biosimilars are reflective of marginal costs of production. If prices exceed marginal costs, this won't modify realized population net health effects which are influenced by prices paid rather than underlying costs. In this scenario total value will be higher than estimated here with additional value accruing to the originator manufacturer and generic/biosimilar supply chain.

Many pharmaceutical products are used and appraised for multiple indications. This raises the question of whether losses in population health in one indication could be justified by gains in another. Under indication‐based pricing, a separate pricing decision is made for each indication, and it is unclear what considerations would support pricing that results in a loss in population health in any individual indication. Where a single price applies across indications we might expect net health gains in secondary indications if the price set for the first indication represents a “ceiling”. However, there is no guarantee that this will be the case if secondary indications offer lower value and/or approval norms exceed measures of opportunity cost. Of the five secondary indications within our case studies, four of the approvals were expected to result in population health losses. There may also be health gains if price discounts in secondary indications apply to earlier indications as observed in two of the case studies considered. Nonetheless, there seems to be no clear rational for setting prices at a level that reduces population health in early indications on the basis that this could be offset by gains from later indications. The secondary indications and price reductions may never materialize, and high prices for early indications may disincentive launches in secondary indications.

In the UK there are Voluntary and Statutory Schemes through which branded medicine manufacturers pay a share of their sales back to the NHS to help control growth in NHS branded medicine spends. The amount paid back varies by year (depending on forecast and actual growth in sales and exemptions) but it is typically 5%–10% of sales (Department of Health & Social Care, 2019; Ferraro et al., 2018). Inclusion of this magnitude of repayments does not modify value shares markedly or alter our conclusions.

### 5.2 Implications for pricing of pharmaceuticals

An important consideration is whether current payment levels can be justified on the basis that they spur innovation, delivering more products and health gains in the future. Long‐term net health effects driven by these dynamic effects are as legitimate as those driven by static considerations (i.e., net health gains from existing drugs). However, pricing that results in *losses* to population health cannot be dynamically efficient. This level of pricing incentivizes the development of future innovations at prices which will also reduce overall health. It would be better for population health if products that can only be incentivized by this level of pricing were not brought to market.

Conversely, a policy that allocates minimal value to manufacturers would stall product development. To determine the appropriate pricing level between allocating 0% and 100% of value to manufacturers, it is necessary to account for both the net health implications for products that are approved, as quantified here, and how pricing contributes to the development of new products (Palnoch, 2019; Pandey et al., 2018). Further work should aim to incorporate these dynamic effects. In this work we have focused on individual appraisals, and a single country setting. The dynamic implications of different pricing policies will be determined by the collective implications of international pricing.

When considering the implications of this work for pricing policy, it is also necessary to consider the uncertainty surrounding the realized population net health effects and payments to manufacturers. From the health systems' perspective, higher uncertainty may necessitate lower prices for unconditional approval to be granted, or the use of conditional reimbursement arrangements (Claxton et al, 2012). However, conditional reimbursement increases uncertainty in payments for the manufacturer, and the dynamic implications of this should also be accounted for.

Finally, our work highlights that current pricing and reimbursement policies will share total value differently depending on product characteristics. For example, biologic products for which generic versions are slower to market and more expensive result in less favorable shares for population health, and more favorable shares for manufacturers. Similarly, products targeting indications with large numbers of prevalent cases, with short treatment durations, or additional costs result in less favorable shares for population health and more favorable shares for manufacturers. This suggests that current value‐based pricing is not directly aligned with total lifetime value and that differential approval norms or payments based on shares of total value may provide a clearer signal of value.

### 5.3 Limitations

Aspects of our model were challenging to parameterize due to a lack of publicly available data relating to patent protection, and availability, pricing and market shares for generic/biosimilar products. Given the importance of this information in assessing the value delivered by pharmaceuticals, and the performance of the UK generics and biosimilars markets, a more concerted effort to make these data publicly available is warranted.

The conclusions presented here rest on an assessment that the approval norms used in reimbursement processes exceed measures of opportunity cost. There is a growing body of evidence that supports this conclusion (Claxton et al., 2015; Lomas et al., 2019; Ochalek et al., 2018) though the exact value for both approval norms and estimates of opportunity cost are subject to ongoing research and vary internationally.

We assumed for simplicity that the measure of opportunity cost would remain constant over time. Data for the period 2003‐2012 suggest that the measure of opportunity cost has been increasing in real terms (Lomas et al., 2019). However, forecasting the measure of opportunity cost over time requires an understanding of real growth in health expenditure, health needs and productivity which is beyond the scope of this study, and particularly challenging in a post‐Covid context.^5^

## 6 CONCLUSIONS

In this study we show how to estimate the total potential long‐term value of new branded pharmaceuticals and how this is shared between patients served by health systems and manufacturers. We show that in the UK the use of approval norms that exceed measures of opportunity cost means that many NICE approvals are expected to reduce overall population health even in the long‐run. Further work is required to consider how these findings should be reflected in reimbursement policies.

## CONFLICTS OF INTEREST

None.

## ETHICS STATEMENT

Not applicable.

## Supporting information

Supplementary Material S1

## Data Availability

The data and models underpinning our analysis are provided as supplementary materials.

## ENDNOTES

^1^
The Department of Health and Social Care has adopted £15,000/quality‐adjusted life year (QALY) as the estimate of health opportunity cost in their impact assessments (Department of Health, 2017; Department of Health and Social Care, 2018a, 2018b) based on empirical estimates of the marginal productivity of NHS expenditure; for the latest estimates see Lomas et al. (2019).

^2^
This may be due to increased uptake in existing clinical indications, or expansion to additional clinical indications. If this results in expansion to indications that are less valuable, this could reduce the share of value accruing as population health gains.

^3^
The magnitude of these effects will depend on long‐term rates of discontinuation from treatment.

^4^
The QALY gain can modify the share of value when the approval norm differs from the measure of opportunity cost and there are non‐product costs or savings. When the approval norm exceeds the measure of opportunity cost and there are non‐product costs (savings), larger QALY gains increase (reduce) the share of value accruing as population health gains.

^5^
In a post‐Covid context it is less clear that the measure of opportunity cost will continue to rise, as growth in expenditure may not continue to outstrip growth in needs and productivity.
