# Supplementary material for: Estimating the shares of the value of branded pharmaceuticals accruing to manufacturers and to patients served by health systems
Source: Health Econ. 2021 Aug 2;30(11):2649–66. doi: 10.1002/hec.4393 (PMC9291963; doi:10.1002/hec.4393)
Supplement: Supplementary file 1 — Supplementary Material S1 [file HEC-30-2649-s001.docx]

Supplementary material contents

[Appendix A: Reflecting non-product related costs 2](#_Toc51245689)

[Appendix B: Data extraction from NICE appraisals 3](#_Toc51245690)

[Appendix C: Time taken for generic/biosimilar products to reach the market 10](#_Toc51245691)

[Appendix D: Cost of generic and biosimilar medicines 12](#_Toc51245692)

[Appendix E: Methods and results of primary care sensitivity analysis 18](#_Toc51245693)

[Appendix F: Implications of faster access and reduced prices for biosimilar and generic products 19](#_Toc51245694)

[Appendix G: Reflecting the consumption value of health 21](#_Toc51245695)

# Appendix A: Reflecting non-product related costs

Incremental non-product costs to the NHS ($\Delta npc$) modify the value accruing to each party.

Total potential net health effects and realised population net health effects in the post-patent period decrease with additional NHS costs, and increase with NHS savings:

$$\begin{aligned} \Delta h-\frac{\Delta npc}{k}-\frac{\Delta mc}{k}\# \left( A.1 \right) \end{aligned}$$

The corresponding total discounted potential net health effects is:

$$\begin{aligned} \sum_{t=1}^{T=\infty} \frac{n_{t}}{{(1+r)}^{t}}\left( \Delta h-\frac{\Delta npc}{k}-\frac{\Delta mc}{k} \right)\#\left( A.2 \right) \end{aligned}$$

Within the patent-period the realised population net health effects is unaffected by NHS costs or savings:

$$\begin{aligned} \Delta h-\frac{\Delta npc}{k}-\left( \Delta h\cdot\frac{\lambda}{k}-\frac{\Delta npc}{k} \right)\#\left( A.3 \right) \end{aligned}$$

The non-product cost terms cancel out as the incremental costs are deducted from the manufacturer price or cost savings are added to the manufacturer price.

The total lifetime realised population net health effects is:

$$\begin{aligned} \sum_{t=1}^{T=t_{p}} \frac{n_{t}}{\left( 1+r \right)^{t}}\left( \Delta h-\Delta h\cdot\frac{\lambda}{k} \right)+\sum_{t=t_{p}+1}^{T=\infty} \frac{n_{t}}{\left( 1+r \right)^{t}}\left( \Delta h-\frac{\Delta npc}{k}-\frac{\Delta mc}{k} \right)\#\left( A.4 \right) \end{aligned}$$

NHS costs decrease the health foregone due to payments to manufacturers, and savings increase the health foregone:

$$\begin{aligned} \Delta h\cdot\frac{\lambda}{k}-\frac{\Delta npc}{k}-\frac{\Delta mc}{k}\#\left( A.5 \right) \end{aligned}$$

The total lifetime health foregone due to payments to manufacturers is:

$$\begin{aligned} \sum_{t=1}^{T=t_{p}} \frac{n_{t}}{\left( 1+r \right)^{t}}\left( \Delta h\cdot\frac{\lambda}{k}-\frac{\Delta npc}{k}-\frac{\Delta mc}{k} \right) \#\left( A.6 \right) \end{aligned}$$

# Appendix B: Data extraction from NICE appraisals

The work by Pennington et al. describing the impact of using the EQ-5D-5L which provided the source for our case studies included 20 appraisals. Of these we were able to include 12 within our analysis. Table B.1 shows the list of included appraisals, and Table B.2 the list of excluded appraisals with reasons for exclusion. Appraisals were excluded because the ICERs used to inform NICE’s final decision making were unclear or did not reflect actual prices paid by the NHS, or because the technology under appraisal was not a drug. For three of the case studies (TA228, 316, and 391), not all subgroups could be reflected within our analysis due to a lack of available data, further detail on the reasons for this is provided in the exclusions table.

Data were extracted from the available appraisal documentation including FAD, academic review group reports, manufacturer submissions and costing reports/spreadsheets. The approach to data extraction is detailed in Table B.3. The data used to predict the duration of on-patent usage for each drug is provided in Table B.4.

**Table B.1: List of included appraisals (ordered by TA number)**

| **TA** | **Drug** | **Recommendation** | **Reference/Source** |
| --- | --- | --- | --- |
| TA228 | Thalidomide *(appraisal partially included – see exclusions table)* | Thalidomide-based combination therapy for first line multiple myeloma if high-dose chemotherapy with stem-cell transplantation is considered inappropriate | <https://www.nice.org.uk/guidance/ta228> |
| TA316 | Enzalutamide *(partially included – see exclusions table)* | Metastatic hormone relapsed prostate cancer previously treated with a docetaxel containing regimen (2+prior cytotoxic regimens) | <https://www.nice.org.uk/guidance/ta316> |
| TA325 | Nalmefene | For people with alcohol dependence: who have a high drinking risk level (defined as alcohol consumption of more than 60 g per day for men and more than 40 g per day for women, according to the World Health Organization’s drinking risk levels) without physical withdrawal symptoms, and who do not require immediate detoxification. | <https://www.nice.org.uk/guidance/ta325> |
| TA335 | Rivaroxaban | In combination with aspirin plus clopidogrel or aspirin alone, for preventing atherothrombotic events in people who have had an acute coronary syndrome with elevated cardiac biomarkers. | <https://www.nice.org.uk/guidance/ta335> |
| TA352 | Vedolizumab | Treating moderately to severely active Crohn’s disease only if: a tumour necrosis factor-alpha inhibitor has failed (that is the disease has responded inadequately or has lost response to treatment) or a tumour necrosis factor-alpha inhibitor cannot be tolerated or is contra-indicated. | <https://www.nice.org.uk/guidance/ta352> |
| TA357 | Pembrolizumab | Treating advanced (unresectable or metastatic) melanoma in adults only: after the disease has progressed with ipilimumab and, for BRAF V600 mutation-positive disease, a BRAF or MEK inhibitor | <https://www.nice.org.uk/guidance/ta357> |
| TA367 | Vortioxetine | Treating major depressive episodes in adults whose condition has responded inadequately to 2 antidepressants within the current episode. | <https://www.nice.org.uk/guidance/ta367> |
| TA381 | Olaparib | Treating adults with relapsed, platinum-sensitive ovarian, fallopian tube or peritoneal cancer who have BRCA1 or BRCA2 mutations whose disease has responded to platinum-based chemotherapy only if they have had 3 or more courses of platinum-based chemotherapy. | <https://www.nice.org.uk/guidance/ta381> |
| TA377 | Enzalutamide | Treating metastatic hormone-relapsed prostate cancer: in people who have no or mild symptoms after androgen deprivation therapy has failed, and before chemotherapy is indicated. | <https://www.nice.org.uk/guidance/ta377> |
| TA391 | Cabazitaxel *(partially included – see exclusions table)* | Treatment of prostate cancer in patients previously treated with or unsuitable for abiraterone or enzalutamide | <https://www.nice.org.uk/guidance/ta391> |
| TA392 | Adalimumab | Treating active moderate to severe hidradenitis suppurativa in adults whose disease has not responded to conventional systemic therapy. | <https://www.nice.org.uk/guidance/ta392> |
| TA428 | Pembrolizumab | Treating locally advanced or metastatic PD-L1-positive non-small-cell lung cancer in adults who have had at least one chemotherapy (and targeted treatment if they have an epidermal growth factor receptor [EGFR]- or anaplastic lymphoma kinase [ALK]-positive tumour), only if: pembrolizumab is stopped at 2 years of uninterrupted treatment and no documented disease progression. | <https://www.nice.org.uk/guidance/ta428> |

**Table B.2 List of excluded appraisals with reasons for exclusion (ordered by TA number)**

| **TA** | **Drug** | **Technology recommended and indication** | **Reason for exclusion** | **Reference/Source** |
| --- | --- | --- | --- | --- |
| TA228 | Bortezomib *(partially included – see inclusions table)* | In combination with an alkylating agent and a corticosteroid is recommended as an option for the first-line treatment of multiple myeloma if high-dose chemotherapy with stem cell transplantation is considered inappropriate and the person is unable to tolerate or has contraindications to thalidomide. | Not clear which ICER informed decision making for bortezomib | <https://www.nice.org.uk/guidance/ta228> |
| TA274 | Ranibizumab | Treating visual impairment due to diabetic macular oedema only if the eye has a central retinal thickness of 400 micrometres or more at the start of treatment. | Rapid review so limited information available in public domain | <https://www.nice.org.uk/guidance/ta274> |
| TA279 | Percutaneous  vertebroplasty and  percutaneous balloon  kyphoplasty | Percutaneous vertebroplasty, and percutaneous balloon kyphoplasty without stenting, are recommended as options for treating osteoporotic vertebral compression fractures only in people who have severe ongoing pain after a recent, unhealed vertebral fracture despite optimal pain management and in whom the pain has been confirmed to be at the level of the fracture by physical examination and imaging. | Medical technology | <https://www.nice.org.uk/guidance/ta279> |
| TA316 | Enzalutamide *(partially included – see inclusions table)* | Metastatic hormone relapsed prostate cancer previously treated with a docetaxel containing regimen (1 prior cytotoxic regimen) | Incremental costs and QALYs not reported for the subgroup of individuals who had received 1 prior cytotoxic regimen | <https://www.nice.org.uk/guidance/ta316> |
| TA363 | Ledpasvir-sofosbuvir | Treating chronic hepatitis C in adults | ICERs do not reflect prices paid by Department for Health and Social Care Commercial Medicines Unit | <https://www.nice.org.uk/guidance/ta363> |
| TA366 | Pembrolizumab | Treating advanced (unresectable or metastatic) melanoma that has not been previously treated with ipilimumab | QALY, cost and ICERs on which the final decision was made are confidential in the FAD and ERG report. The QALYs, costs and ICERs in the CS and Pennington report do not reflect PAS discounts for the comparators | <https://www.nice.org.uk/guidance/ta366> |
| TA391 | Cabazitaxel *(partially included – see inclusions table)* | Treating prostate cancer in patients who are suitable for treatment with abiraterone or enzalutamide. | QALYs, costs and ICERs not available for the subgroup of patients suitable for abiraterone or enzalutamide. | <https://www.nice.org.uk/guidance/ta391> |
| TA427 | Pomalidomide | In combination with low‑dose dexamethasone, as an option for treating multiple myeloma in adults at third or subsequent relapse; that is, after 3 previous treatments including both lenalidomide and bortezomib. | Costs, QALYs and ICERs not available for full set of comparators for confidentiality reasons. | <https://www.nice.org.uk/guidance/ta427> |
| HST2 | Elsosulfase alfa | Treating mucopolysaccharidosis type IVa (MPS IVa) | ICER confidential in NICE documents and Pennington et al. reports | <https://www.nice.org.uk/guidance/HST2> |
| Not reported | Intervention for treating relapsing‑remitting multiple sclerosis | Not reported | TA number not revealed in Pennington et al. reports | Not reported |

**Table B.3: Data extracted from NICE appraisals**

| **Parameter** | **Description of data extraction** |
| --- | --- |
| Incremental costs, incremental QALYs and ICERs reflecting the committees final decision | Extracted from the Final Appraisal Determination (FAD) or underlying appraisal documents if available. Otherwise extracted from Pennington et al. (Pennington, Hernandez-Alava, Pudney, & Wailoo, 2018) who had access to the underlying models and were therefore able to reproduce the analyses.  Where the committee’s final decision was based on a range of ICERs reflecting different scenario analyses the average costs and QALYs across scenarios were calculated for each comparator and the incremental cost-effectiveness analysis re-performed using these averages. |
| Incremental NHS costs (other than product acquisition costs) | For technologies approved without a patient access scheme (PAS): NHS costs estimated as the difference between the total incremental costs and the incremental product acquisition cost. Total product acquisition cost was obtained from the manufacturer submission as disaggregated cost data are not typically reported for the analyses that inform final committee decision making.  For technologies approved with a PAS: incremental NHS costs estimated directly from the manufacturer submission and are unlikely to reflect the scenario that informed the final committee decision making.  Where a cost breakdown was not available incremental NHS costs were assumed to be zero.  For TA377 a significant part of the non-product cost savings are driven by reduced use of enzalutamide in the post-chemotherapy setting. This reflected the assumption that if patients received enzalutamide in the pre-chemotherapy setting they would not receive it again later in their care pathway. These cost savings will not persist at the same level once enzalutamide goes off patent. We therefore assumed that once generic enzalutamide became available the cost savings reduce to £4,051. This was calculated assuming zero savings in the post-progression 2 health state once enzalutamide had gone off patent. |
| Treatment duration | Mean treatment duration used where available, medians used otherwise.  We assume that all patients initiating treatment on a branded product complete treatment with the branded product and all patients initiating treatment with a generic product complete treatment with the generic product. In practise, some patients who initiate treatment before a generic product becomes available will switch to the generic product during the course of their treatment. However, as the case studies considered have relatively short treatment durations we do not expect that relaxing this assumption would substantively impact our results. |
| Whether treatment is additive or substitute for existing interventions | Assessed using NICE FAD description of technology. |
| Number of patients expected to receive treatment | Extracted from NICE costing report if available. Otherwise extracted from academic group report or company submission. In some instances the appraisal documentation provided limited information on patient population size and additional data was sought from the literature. |
| Location of prescribing | Determined from appraisal documentation and review by a pharmacist. |

Notes: FAD=final appraisal determination; ICER = incremental cost-effectiveness ratio; PAS = patient access scheme; QALY=quality-adjusted life years.

**Table B.4 Data relating to period of patent protection for case study appraisals**

| **TA #** | **Product** | **Disease** | **Years from first marketing authorisation to FAD issue^1^** | **Additional years of availability via CDF^2^** | **Predicted period of on-patent usage^3^** |
| --- | --- | --- | --- | --- | --- |
| 325 | Nalmefene | Alcohol dependence | 2 | 0 | 11 |
| 367 | Vortioxetine | Major depressive episodes | 2 | 0 | 11 |
| 335 | Rivaroxaban | Acute coronary syndrome | 6 | 0 | 7 |
| 228 | Thalidomide | Multiple myeloma | Not applicable | Not applicable | 8^4^ |
| 392 | Adalimumab | Hidradenitis Suppurativa | Not applicable | Not applicable | 3^4^ |
| 352 | Vedolizumab | Crohn's disease | 1 | 0 | 12 |
| 377 | Enzalutamide | Prostate cancer (pre-chemotherapy) | 2 | 1 | 12 |
| 428 | Pembrolizumab | NSCLC | 1 | 0 | 12 |
| 391 | Cabazitaxel | Prostate cancer | 5 | 3 | 11 |
| 316 | Enzalutamide | Prostate cancer (post-chemotherapy) | 1 | 1 | 13 |
| 357 | Pembrolizumab | Melanoma | 0 | 0 | 13 |
| 381 | Olaparib | Ovarian, fallopian tube and peritoneal cancer | 1 | 0 | 12 |

Notes: FAD = final appraisal determination; CDF = cancer drugs fund.

^1^ The date of marketing authorisation was obtained from the company submission.

^2^ Dates of availability for drugs via the Cancer Drugs Fund were obtained from the national archives.(NHS, 2014)

^3^ The predicted period of on-patent usage is calculated using the average time from marketing authorisation to loss of exclusivity (13 years as documented in Table 6 of the main text), and subtracting from this time from first marketing authorisation to FAD issue. If prior to the FAD there were additional years of availability via the CDF these were added back on to the period of on-patent usage.

^4^ Dates of loss of patent protection were available for these two drugs.(UK Medicines Information, 2012, 2013, 2014, 2015, 2016) These were used directly alongside the FAD date to calculate the period of on-patent usage.

# Appendix C: Time taken for generic/biosimilar products to reach the market

As discussed in the main text, the European Commission Pharmaceutical Sector Inquiry 2009 report provided data on time from loss of exclusivity to availability of first generic for a sample of small-molecule drugs used in the community. In this analysis, time to generic entry estimates were pooled across European countries and different drugs resulting in 1,085 observations for analysis. This information was presented as a Kaplan Meier curve with follow-up of up to 8 years. We used a published algorithm to recover pseudo individual product data from the Kaplan Meier curve for analysis.(Guyot, Ades, Ouwens, & Welton, 2012)

Comparable UK data were only reported at 1 year follow-up in this report. The 1 year survival estimates for the UK and Europe were, therefore, used to estimate a hazard ratio describing the increased rate of generic entry in the UK compared to Europe as a whole. This hazard ratio was estimated as 1.73, indicating that the rate of generic entry was 73% higher in the UK than in Europe. The hazard ratio was applied to the European time to generic entry survival curve to obtain a calibrated UK survival curve.

There is no publicly accessible list of approved biologic or biosimilar products. We therefore developed a new dataset using publicly available reports. A list of biologic drugs expected to lose exclusivity and the estimated date for loss of exclusivity were obtained from Prescribing Outlook documents 2012-2016.(UK Medicines Information, 2012, 2013, 2014, 2015, 2016) These reports are produced by UK Medicines Information to inform budget setting, prescribing planning and medicines management. The dates provided are *predicted* dates taking in to account the Supplementary Protection Certificate (SPC) and paediatric extensions. They may, therefore, differ from the exact date the biologic loses exclusivity in some cases. For each biologic expected to lose exclusivity, the British National Formulary (BNF) was checked in October 2019 to identify whether a biosimilar medicine was now available.(National Institute for Health and Care Excellence) For products for which a biosimilar has entered the market, the date on which the first biosimilar product was available was estimated based on the Prescribing Outlook documents or NHS Commissioning Framework documents,(NHS, 2017) and again represents an estimated date. Where a date was not available for a specific biosimilar, the mid-point between the last available follow up and immediate entry was used to inform the analysis. Thirty-four drugs were included in the sample and the data available is shown in Table C.1.

We fitted parametric survival models to both the small-molecule and biologic datasets to facilitate extrapolation beyond the observed data. We followed guidance from the NICE Technical Support(Latimer, 2013) document and fitted a range of parametric survival models: exponential, Weibull, Gompertz, log-logistic, lognormal and generalised gamma. Model fit was assessed according to Akaike’s Information Criteria (AIC), log-cumulative hazard plots, hazard plots, and visual assessment of the concordance between model predictions and Kaplan Meier plots. No external data were available to support selection of the models; however, the extrapolations were reviewed visually to identify any extrapolations considered implausible based on the study team’s judgement. Based on this, the Weibull model was selected for the small molecule and the log-normal for the biologics/biosimilars analysis.

**Table C.1: Dates of biologic loss of patent protection and biosimilar entry**

| **Biologic** | **Patent expiry date** | **Biosimilar available** | **First biosimilar availability date in UK** |
| --- | --- | --- | --- |
| Somatropin | 01/06/2002 | Yes | Date not available |
| Eptifibatide | 01/01/2012 | Yes | Date not available |
| Basiliximab | 01/04/2013 | No |  |
| Rituximab | 01/11/2013 | Yes | 01/04/2017 |
| Alemtuzumab | 01/02/2014 | Yes | Date not available |
| Somatropin^1^ | 01/03/2014 | Yes | 01/07/2016 |
| Anakinra | 01/05/2014 | No |  |
| Trastuzumab | 01/07/2014 | Yes | 01/07/2015 |
| Palivizumab | 01/08/2014 | No |  |
| Cetuximab | 01/09/2014 | No |  |
| Panitumumab | 01/09/2014 | No |  |
| Pertuzumab | 01/12/2014 | No |  |
| Infliximab | 01/02/2015 | Yes | 01/07/2015 |
| Glatiramer | 01/05/2015 | Yes | Date not available |
| Insulin glargine | 01/05/2015 | Yes | 01/09/2015 |
| Insulin aspart biphasic | 01/06/2015 | No |  |
| Tenecteplase | 01/06/2015 | No |  |
| Etanercept | 01/07/2015 | Yes | 01/04/2016 |
| Rasburicase | 01/07/2015 | No |  |
| Botulinum Toxin | 01/01/2016 | No |  |
| Darbepoetin alfa | 01/06/2016 | No |  |
| Agalsidase alfa | 01/08/2016 | No |  |
| Agalsidase beta | 01/08/2016 | No |  |
| Agalsidase beta | 01/08/2016 | No |  |
| Velaglucerase alfa | 01/01/2017 | No |  |
| Peginterferon alfa | 01/05/2017 | No |  |
| Omalizumab | 01/08/2017 | No |  |
| Pegfilgrastim | 01/08/2017 | Yes | 01/08/2017 |
| Pegvisomant | 01/11/2017 | No |  |
| Abatacept | 01/12/2017 | No |  |
| Enfuvirtide | 01/04/2018 | No |  |
| Adalimumab | 01/10/2018 | Yes | 01/10/2018 |
| Insulin detemir | 01/05/2019 | No |  |
| Insulin glulisine | 01/09/2019 | No |  |

Notes: vaccines removed from analysis, three drugs excluded as no BNF data available on biosimilar entry.

# Appendix D: Cost of generic and biosimilar medicines

Due to a lack of data in the literature relating to the cost of small-molecule generic drugs used within hospitals, we conducted an analysis based on publicly available data. A sample of drugs was generated by identifying drugs that had been subject to a NICE technology appraisal in the period 2000/01-2003/04. This time frame was chosen so that the majority of drugs would now be off-patent and have generics available. Drugs were included if they were small-molecule, had a generic product available, the current price for the generic product was available via eMIT 2017-2018(Department of Health and Social Care, 2017), regimen and cost data were provided in the original technology appraisal, and the products were expected to be prescribed in a hospital setting (at least initially). eMIT is the drugs and pharmaceutical electronic market information tool and provides information on the price paid by English trusts for pharmaceuticals bought by hospitals.

In total, 16 drugs were included in the analysis. We extracted from each HTA the details of the dosage and regimen for the treatment and the original branded cost for all formulations and package sizes listed. The corresponding generic prices for each pack were obtained from eMIT. The most cost efficient means of generating the required dosage was then calculated. Where a range of doses is reported, the average cost across doses was used. Unless stated otherwise, calculations based on body surface area assumed 1.75m^2^. Costing assumes wastage if total vial dose is larger than the required dose.

The data used in this analysis are presented as Table D.1. An average monthly cost across these appraisals was calculated separately for tablets/capsules and vials as these were expected to have different costs of production.

**Table D.1: Data included in analysis of generic prices for drugs used in hospitals**

| **TA ID** | **Technology** | **Pack size for brand** | **Brand price** | **Units in pack**  **in eMIT** | **Generic price** | **Dose** | **Assumed packs used** | **Branded monthly cost** | **Generic monthly cost** |
| --- | --- | --- | --- | --- | --- | --- | --- | --- | --- |
| TA003 | Paclitaxel | 30mg (1 vial) | 124.79 | 1 vial | 8.62 | 175 mg/m^2^ (Total dose of 306mg)  Dose: every 3 weeks | 3 x 100mg and 1 x 30 mg | £1800.57 | £53.56 |
|  |  | 100mg (1 vial) | 374 | 1 vial | 9.49 |  |  |  |  |
| TA006 | Docetaxel | 20mg (1 vial) | 175 | 1 vial | 11.61 | 100 mg/m^2^ (Total dose of 175mg)  Dose: every 3 weeks | 2 x 80mg and 1 x 20mg | £1913.52 | £99.03 |
|  |  | 80mg (1 vial) | 575 | 1 vial | 28.48 |  |  |  |  |
| TA019 | Donepezil | 10mg (28 tablets) | 95.76 | 28 tablets | 0.46 | 10 mg  Dose: per day |  | £89.12 | £0.46 |
|  |  | 5mg (28 tablets) | 68.32 | 28 tablets | 0.39 | 5 mg  Dose: per day |  |  |  |
| TA019 | Rivastigmine^1^ | 3mg (56 tablets) | 63 | 28 tablets | 1.43 | 3mg twice per day | 2 x 3mg | £68.44 | £3.98 |
|  |  | 6mg (56 tablets) | 63 | 28 tablets | 2.23 | 6mg twice per day | 2 x 6mg (6 x 3mg for brand) |  |  |
| TA020 | Riluzole | 50mg (56 tablets) | 286 | 56 tablets | 17.23 | 50mg  Dose: twice/day | 2 x 50mg tabs | £310.68 | £18.72 |
| TA023 | Temozolomide | 5mg (5 tablets) | 17.3 | 5 tablets | 2.18 | 340 mg  Dose: per day. Administered as a 5 day cycle every 28 days. | 3 x 100mg and 2 x 20mg (Branded)  4*100mg (generic) | £1274.43 | £44.76 |
|  |  | 20mg (5 tablets ) | 69.2 | 5 tablets | 9.47 |  |  |  |  |
|  |  | 100mg (5 tablets ) | 346 | 5 tablets | 10.33 |  |  |  |  |
|  |  | 250mg (5 tablets ) | 865 | 5 tablets | 47.32 |  |  |  |  |
| TA026 | Gemcitabine (1st line)^2^ | 200mg (1 vial) | 32.55 | 1 vial | 3.12 | 800mg/m^2^ (Total dose of 1360mg)  Dose: once weekly for 3 weeks followed by 1 week break | 1g+2 x 200mg | £952.13 | 61.19 |
|  |  | 1g (1 vial) | 162.76 | 1 vial | 8.66 | 1000 mg/m^2^ (Total dose of 1700mg)  Dose: once weekly for 3 weeks followed by 1 week break | 1g+4 x 200mg |  |  |
|  |  |  |  |  |  | 1250 mg/m^2^ Total dose of 2125mg)  Dose: once weekly for 3 weeks followed by 1 week break | 2 x 1g+200mg |  |  |
| TA026 | Vinorelbine (1st line)^2^ | 1ml, 10mg/ml  (1 vial) | 31.25 | 10 vials | 35.83 | 25 mg/m^2^ (Total dose of 42.5mg)  Dose: Once a week | 5ml vial | £704.97 | £40.65 |
|  |  | 5ml, 50mg/ml  (1 vial) | 147.06 | 10 vials | 75.90 | 30 mg/m^2^ Total dose of 51mg)  Dose: Once a week | 5ml + 1ml vial |  |  |
| TA027 | Celecoxib | 100mg (60 tablets) | 18.34 | 60 tablets | 1.15 | 200 mg  Dose: per day | 2 x 100mg | £27.89 | £1.75 |
|  |  |  |  |  |  | 400 mg  Dose: per day | 4 x 100mg |  |  |
| TA029 | Fludarabine - Intravenous formulation (2nd line)^5^ | 50mg (1 vial) | 130 | 1 vial | 76.6 | 25mg/m^2^  (Total dose of 42.5mg)  Dose: per daily for 5 consecutive days in every 28 days | 1 x 50mg | £704.17 | £414.72 |
| TA045 | Pegylated liposomal doxorubicin hydrochloride (PLDH) | 10ml 2mg/ml (1 vial) | 411.3 | NA | NA | 50 mg/m^2^ (Total dose 87.5mg)  Dose: once every 4 weeks | 2 x 50mg/25ml | £1762.56 | £38.52 |
|  |  | 25ml 2mg/ml (1 vial) | 813.49 | 1 pack | 17.78 |  |  |  |  |
| TA050 | Imatinib^3^ | 100mg (1 tablet) | 12.98 | 60 tablets | 61.33 | 400 mg  Dose: per day^4^ | 4 x 100mg | £1974.04 | £155.45 |
|  |  |  |  |  |  | 600 mg  Dose: per day^4^ | 6 x 100mg |  |  |
| TA062 | Capecitabine^2^ | 150mg (60 tablets) | 44.46 | 60 tablets | 8.15 | 1250 mg/m^2^  Dose: twice/day for 14 days followed by 7 day rest period (Total daily dose of 4250mg) | 9 x 500mg | £448.65 | £40.62 |
|  |  | 500mg (120 tablets) | 295 | 120 tablets | 26.71 |  |  |  |  |
| TA076 | Gabapentin^5^ | per/mg cost | 0.0016 | 100 tablets (100mg) | 1.04 | 900 mg  Dose: per day (Recommended minimum dose) | 3 x 300mg (generic only) | £80.30 | £2.81 |
|  |  |  |  | 100 tablets (300mg) | 1.68 | 2400 mg  Dose: per day (Recommended maximum dose) | 8 x 300mg (generic only) |  |  |
|  |  |  |  | 100 tablets (400mg) | 2.39 |  |  |  |  |
|  |  |  |  | 100 tablets (600mg) | 5.53 |  |  |  |  |
|  |  |  |  | 100 tablets (800mg) | 9.15 |  |  |  |  |
| TA076 | Topiramate^5^ | per/mg cost | 0.0108 | 15mg (60 tablets) | 8.94 | 400 mg  Dose: per day  (Recommended minimum dose) | 4 x 100mg (generic only) | £197.10 | £25.22 |
|  |  |  |  | 25mg (60 tablets) | 3.13 | 800 mg  Dose: per day (Recommended maximum dose) | 8 x 100mg (generic only) |  |  |
|  |  |  |  | 50mg (60 tablets ) | 7.70 |  |  |  |  |
|  |  |  |  | 100mg (60 tablets ) | 8.29 |  |  |  |  |
|  |  |  |  | 200mg (60 tablets) | 21.60 |  |  |  |  |
| TA076 | Levetiracetam^5^ | per/mg cost | 0.0016 | 250mg (60 tablets) | 3.07 | 1000mg Dose: per day (Recommended minimum dose | 1 x 1g (generic only) | £97.33 | £7.10 |
|  |  |  |  | 500mg (60 tablets) | 4.69 | 3000mg Dose: per day (Recommended maximum dose) | 3 x 1g (generic only) |  |  |
|  |  |  |  | 750mg (60 tablets) | 6.62 |  |  |  |  |
|  |  |  |  | 1g (60 tablets) | 7.00 |  |  |  |  |

Notes^:^

^1^ Rivastigmine is given twice a day starting with a low dose of 3mg per day and increasing to between 6 and 12 mg per day. We use the long-term dosage here.

^2^ The dosage here is calculated based on body surface area of 1.7m^2^. This is the approach used in the HTA report.

^3^ Pack size not stated in HTA. Only the price for 100mg is provided.

^4^ Recommended dosage is 400mg/day for those in chronic phase and 600mg/day for those in accelerated phase and blast phase.

^5^ For Gabapentin, Topiramate and Levetiracetam : the cost per mg was provided in the HTA but EMIT provides cost for multiple pack sizes and product strengths. As mentioned above, the most cost-efficient combination of strengths was taken from EMIT

For biosimilar medicines, data were obtained from publicly available tendering documents. These were available for two drugs – adalimumab and infliximab.

For adalimumab, the following reference prices were supplied to clinical commissioning groups (CCGs) for the financial year 2019/2020 (values represent regional groups other than South London):(NHS, 2019)

- Adalimumab 20mg £1775.50 per patient per year (pro rata) including homecare costs; and
- Adalimumab 40mg £3550.00 per patient per year (pro rata) including homecare costs.

These represent the amounts that trusts charge commissioners for adalimumab. The majority of patients receiving adalimumab will receive the 40mg maintenance dose and homecare costs are not expected to be substantial, so these data were considered a reasonable approximation of the cost of acquiring adalimumab or adalimumab biosimilars.

The NHS Commissioning framework for biological medicines(NHS, 2017) reported a price drop of 59% for infliximab and infliximab biosimilars following the entry of biosimilar products. We calculated the original branded price for infliximab using data from previous NICE appraisals(National Institute for Health and Care Excellence, 2008, 2010a, 2010b, 2016a, 2016b) which reported total annual costs for different indications as shown in Table D.2. We used an average of these costs (removing administration costs) and applied the 59% discount in order to estimate the cost of infliximab and infliximab biosimilars as shown in Table D.2.

The average of the annual cost of adalimumab/biosimilar (£3,550) and infliximab/biosimilar (£4,356) was used to calculate the average annual cost of biologics and biosimilars following biosimilar availability.

**Table D.2: Annual cost of branded infliximab**

| **TA #** | **Indication** | **Year 1** | **Subsequent years** |
| --- | --- | --- | --- |
| TA383 | Ankylosing spondylitis and non-radiographic axial spondyloarthritis | £15,107 | £12,589 |
| TA375 | Rheumatoid Arthritis | £10,071 | £8,812 |
| TA199 | Psoriatic arthritis | £12,742 | £10,352 |
| TA187 | Crohn's disease | £10,839 | £8,807 |
| TA134 | Psoriasis | £13,500 | £10,910 |
| Average | | £12,452 | £10,294 |
| TA187 | Administration costs^1^ | £2,064 | £1,677 |
| Average excluding administration costs^2^ | | £11,626 | £9,623 |
| Applying 59% discount to reflect reduction in cost following availability of biosimilars | | £4,767 | £3,945 |

^1^ Cost of infusion from TA187, annual cost based on 8 infusions in year 1 and 6.5 in subsequent years.

^2^ Administration costs removed from TA199 and TA187, costings from other appraisals did not include administration costs.

# Appendix E: Methods and results of primary care sensitivity analysis

Three of the case study products may be prescribed in the primary care setting (nalmefene, vortioxetine, and rivaroxaban). A sensitivity analysis was conducted using data relating to the use and pricing of generic drugs in the primary care setting.

For small-molecule drugs used in primary care, uptake of generics has been estimated as 40% in the first year the generic is available and 49% in the second year. This is based on data from 128 top-selling small molecule drugs prescribed in the community that lost exclusivity in 2000-2007.(European Commission, 2009) In the absence of longer-term data, generic uptake is assumed to reach 100% in the third year following generic availability.

Data on pricing of generic and branded products following availability of generic versions of the product are also available from this source. These data should be viewed with caution as they are based on IMS prices which likely overestimate prices paid by the NHS.(Ferraro, O'Neill, & Towse, 2018) The data available for the UK indicated a price drop for the branded product of 10% in the first year of generic availability and 18% in the second year. Generic products were 22% cheaper than the on-patent brand price in the first year of generic entry and 43% cheaper in the second year.

To extrapolate these estimates we assume a linear-log relationship between percentage price drop and time. This predicted price discounts relative to the original brand price of 38% for the originator and 95% for generics at 10 years following generic entry. We assumed that beyond this time point, no further price drop would occur and that the 10 year price represents marginal costs. This analysis therefore assumes that generic manufacturers price above marginal cost in the short term. This means that the shares of value no longer sums to 100%, as some surplus is now appropriated by generic manufacturers.

**Table E.1: Surplus estimates for drugs that may be prescribed in primary or secondary care**

| Product | FAD ICER  (£/QALY) | Total potential net health gains  (net QALYs) | Realised population net health effects  (net QALYs) | Health foregone due to payments to manufacturers (net QALYs) | Share of value accruing as population health gains | Share of value accruing to manufacturer |
| --- | --- | --- | --- | --- | --- | --- |
| Nalmefene  – secondary care | 1,110 | 80,460 | 73,188 | 7,271 | 91% | 9% |
| Nalmefene  – primary care | 1,110 | 97,420 | 81,675 | 12,809 | 84% | 13% |
| Vortioxetine  – secondary care | 2,970 | 37,758 | 35,165 | 2,593 | 93% | 7% |
| Vortioxetine  – primary care | 2,970 | 37,758 | 34,975 | 2,783 | 93% | 7% |
| Rivaroxaban  – secondary care | 5,622 | 30,605 | 28,774 | 1,831 | 94% | 6% |
| Rivaroxaban  – primary care | 5,622 | 37,522 | 32,791 | 3,545 | 87% | 9% |

# Appendix F: Implications of faster access and reduced prices for biosimilar and generic products

We explored the impact of having immediate access to generic/biosimilar products and their prices dropping by 25%.

We present results for this analysis in Table F.1, and in Figure F.1, using the same format as Figure 4 in the main manuscript.

**Table F.1: Shares of value assuming marked improvements to access and pricing of biosimilars and generics**

| Product | FAD ICER  (£/QALY) | Total potential net health gains  (net QALYs) | Realised population net health effects  (net QALYs) | Health foregone due to payments to manufacturers (net QALYs) | Share of value accruing as population health gains | Share of value accruing to manufacturer |
| --- | --- | --- | --- | --- | --- | --- |
| Nalmefene - sc | 1,110 | 85,228 | 78,046 | 7,182 | 92% | 8% |
| Vortioxetine - sc | 2,970 | 37,758 | 35,704 | 2,054 | 95% | 5% |
| Rivaroxaban - sc | 5,622 | 32,525 | 30,835 | 1,690 | 95% | 5% |
| Thalidomide | 9,174 | 69,087 | 59,060 | 10,027 | 85% | 15% |
| Adalimumab | 19,328 | 2,215 | 487 | 1,728 | 22% | 78% |
| Vedolizumab | 21,620 | 1,518 | 115 | 1,403 | 8% | 92% |
| Enzalutamide_prechemo | 32,985 | 121,161 | 38,638 | 82,522 | 32% | 68% |
| Pembrolizumab_NSCLC | 44,490 | 17,730 | -8,085 | 25,816 | -46% | 146% |
| Cabazitaxel | 45,159 | 2,510 | 50 | 2,460 | 2% | 98% |
| Enzalutamide_postchemo | 45,626 | 1,552 | -694 | 2,246 | -45% | 145% |
| Pembrolizumab_Melanoma | 46,662 | 4,035 | -1,736 | 5,771 | -43% | 143% |
| Olaparib | 46,973 | 4,388 | -948 | 5,336 | -22% | 122% |

Figure F.1: Range of shares of value for (a) small molecule and (b) biologic drugs for different approval norms with improved access to and pricing for generic/biosimilar products.

# Appendix G: Reflecting the consumption value of health

We consider how our results change if we value total potential net health gains generated by the new medicine and realised population net health effects in consumption terms. We use two values for the consumption value of health (v). A value of 60,000/QALY is used to reflect the value used by the UK department of health.(HM Treasury, 2018) A value of £30,000/QALY is also used as this might be considered a reasonable value based on available literature.(Ryen & Svensson, 2015; Thokala, Ochalek, Leech, & Tong, 2018; Vallejo-Torres et al., 2016) Payments to manufacturers are assumed to be used directly or indirectly for consumption.

We illustrate this in the first instance using a numeric example where the product generates a potential 2,000 QALYs. Payments to the manufacturer are £15 million resulting in opportunity costs of 1,000 QALYs. The remaining 1,000 QALYs is accrued as population health gains (i.e. value is shared 50%-50% when everything is valued in terms of net health effects). Using £60,000/QALY as the consumption value of health, the consumption value of the total available net health gains is £120 million (£60,000/QALY x 2,000 QALYs). The consumption value of the realised population health gains is £60 million (£60,000/QALY x 1,000). The same share of value (50%) is accrued as population health gains when everything is valued in consumption terms. The manufacturer, however, accrues only 12.5% of the consumption value of the potential health gains (£15 million divided by £120 million). The total value generated in consumption terms is only 62.5% of the consumption value of the potential net health gains from the new medicine. This is because the money paid to the manufacturer and assumed to be used for consumption (£15 million) could have generated consumption value at a rate of v/k if retained in the health system and used to generate health.

The results for the case studies are shown in Table G.1 and Table G.2.

**Table G.1: Value in consumption terms (consumption value of health=£30,000/QALY)**

| Product | FAD ICER  (£/QALY) | Total potential net health gains  (net QALYs) | Realised population net health effects  (net QALYs) | Health foregone due to payments to manufacturers (net QALYs) | Share of value accruing as population health gains | Share of value accruing to manufacturer |
| --- | --- | --- | --- | --- | --- | --- |
| Nalmefene - sc | 1,110 | £2,413,787,052 | £2,195,645,905 | £109,070,573 | 91% | 5% |
| Vortioxetine - sc | 2,970 | £1,132,748,317 | £1,054,959,096 | £38,894,611 | 93% | 3% |
| Rivaroxaban - sc | 5,622 | £918,146,395 | £863,216,800 | £27,464,798 | 94% | 3% |
| Thalidomide | 9,174 | £2,063,152,620 | £1,670,789,170 | £196,181,725 | 81% | 10% |
| Adalimumab | 19,328 | £61,150,894 | -£8,760,574 | £34,955,734 | -14% | 57% |
| Vedolizumab | 21,620 | £29,725,075 | -£12,951,777 | £21,338,426 | -44% | 72% |
| Enzalutamide  _prechemo | 32,985 | £3,732,313,008 | £785,289,842 | £1,473,511,583 | 21% | 39% |
| Pembrolizumab  _NSCLC | 44,490 | £490,788,719 | -£783,786,737 | £637,287,728 | -160% | 130% |
| Cabazitaxel | 45,159 | £75,299,487 | -£13,683,403 | £44,491,445 | -18% | 59% |
| Enzalutamide  _postchemo | 45,626 | £45,623,467 | -£31,081,028 | £38,352,247 | -68% | 84% |
| Pembrolizumab  _Melanoma | 46,662 | £111,510,199 | -£159,360,581 | £135,435,390 | -143% | 121% |
| Olaparib | 46,973 | £129,499,791 | -£62,332,913 | £95,916,352 | -48% | 74% |

**Table G.2: Value in consumption terms (consumption value of health=£60,000/QALY)**

| Product | FAD ICER  (£/QALY) | Total potential net health gains  (net QALYs) | Realised population net health effects  (net QALYs) | Health foregone due to payments to manufacturers (net QALYs) | Share of value accruing as population health gains | Share of value accruing to manufacturer |
| --- | --- | --- | --- | --- | --- | --- |
| Nalmefene - sc | 1,110 | £4,827,574,103 | £4,391,291,810 | £109,070,573 | 91% | 2% |
| Vortioxetine - sc | 2,970 | £2,265,496,635 | £2,109,918,192 | £38,894,611 | 93% | 2% |
| Rivaroxaban - sc | 5,622 | £1,836,292,791 | £1,726,433,600 | £27,464,798 | 94% | 1% |
| Thalidomide | 9,174 | £4,126,305,240 | £3,341,578,341 | £196,181,725 | 81% | 5% |
| Adalimumab | 19,328 | £122,301,788 | -£17,521,148 | £34,955,734 | -14% | 29% |
| Vedolizumab | 21,620 | £59,450,149 | -£25,903,555 | £21,338,426 | -44% | 36% |
| Enzalutamide  _prechemo | 32,985 | £7,464,626,016 | £1,570,579,684 | £1,473,511,583 | 21% | 20% |
| Pembrolizumab  _NSCLC | 44,490 | £981,577,438 | -£1,567,573,474 | £637,287,728 | -160% | 65% |
| Cabazitaxel | 45,159 | £150,598,974 | -£27,366,807 | £44,491,445 | -18% | 30% |
| Enzalutamide  _postchemo | 45,626 | £91,246,934 | -£62,162,055 | £38,352,247 | -68% | 42% |
| Pembrolizumab  _Melanoma | 46,662 | £223,020,399 | -£318,721,163 | £135,435,390 | -143% | 61% |
| Olaparib | 46,973 | £258,999,581 | -£124,665,827 | £95,916,352 | -48% | 37% |

References

Department of Health and Social Care. (2017). eMIT National Database. Retrieved July 16 2019, from Department of Health and Social Care

European Commission. (2009). *Pharmaceutical Sector Inquiry Final Report*. Retrieved from <https://ec.europa.eu/competition/sectors/pharmaceuticals/inquiry/staff_working_paper_part1.pdf>

Ferraro, J., O'Neill, P., & Towse, A. (2018). *Measurement of Medicines Expenditure in the Context of the 2014-18 PPRS*. Retrieved from

Guyot, P., Ades, A. E., Ouwens, M. J., & Welton, N. J. (2012). Enhanced secondary analysis of survival data: reconstructing the data from published Kaplan-Meier survival curves. *BMC Med Res Methodol, 12*(1), 9. doi:10.1186/1471-2288-12-9

HM Treasury. (2018). The green book: Central government guidance on appraisal and evaluation. *London: HM Treasury*.

Latimer, N. (2013). *NICE DSU Technical Support Document 14: Survival ANalysis for Economic Evaluations Alongside Clinical Trials - Extrapolation with Patient-Level Data*. Retrieved from

National Institute for Health and Care Excellence. British National Formulary. Retrieved from <https://bnf.nice.org.uk/>

National Institute for Health and Care Excellence. (2008). *Infliximab for the treatment of adults with psoriasis*. Retrieved from <https://www.nice.org.uk/guidance/ta134>

National Institute for Health and Care Excellence. (2010a). *Etanercept, infliximab and adalimumab for the treatment of psoriatic arthritis*. Retrieved from <https://www.nice.org.uk/guidance/ta199>

National Institute for Health and Care Excellence. (2010b). *Infliximab and adalimumab for the treatment of Crohn's disease*. Retrieved from <https://www.nice.org.uk/guidance/ta187>

National Institute for Health and Care Excellence. (2016a). *Adalimumab, etanercept, infliximab, certolizumab pegol, golimumab, tocilizumab and abatacept for rheumatoid arthritis not previously treated with DMARDs or after conventional DMARDs only have failed*. Retrieved from <https://www.nice.org.uk/guidance/ta375>

National Institute for Health and Care Excellence. (2016b). *TNF-alpha inhibitors for ankylosing spondylitis and non-radiographic axial spondyloarthritis*. Retrieved from <https://www.nice.org.uk/guidance/ta383>

NHS. (2014). Cancer Drug Fund Summaries. Retrieved from <https://webarchive.nationalarchives.gov.uk/20140721172243/https://www.england.nhs.uk/ourwork/pe/cdf/cdf-drug-sum/>

NHS. (2017). *Commissioning framework for biological medicines (including biosimilar medicines)*. Retrieved from <https://www.england.nhs.uk/wp-content/uploads/2017/09/biosimilar-medicines-commissioning-framework.pdf>

NHS. (2019). Reference prices for Adalimumab. Retrieved from <https://www.england.nhs.uk/wp-content/uploads/2019/04/reference-prices-for-adalimumab-letter.pdf>

Pennington, B., Hernandez-Alava, M., Pudney, S., & Wailoo, A. (2018). *Comparing the EQ-5D-3L and 5L Versions. What are the Implications for Model-Based Cost Effectiveness Estimates*. Retrieved from

Ryen, L., & Svensson, M. (2015). The Willingness to Pay for a Quality Adjusted Life Year: A Review of the Empirical Literature. *Health Econ, 24*(10), 1289-1301. doi:10.1002/hec.3085

Thokala, P., Ochalek, J., Leech, A. A., & Tong, T. (2018). Cost-Effectiveness Thresholds: the Past, the Present and the Future. *Pharmacoeconomics, 36*(5), 509-522. doi:10.1007/s40273-017-0606-1

UK Medicines Information. (2012). *Prescribing Outlook New Medicines - September 2012*. Retrieved from <https://www.ukmi.nhs.uk/filestore/ukmianp/prescribingoutlook-newmedicines2012amendedoct2012.pdf>

UK Medicines Information. (2013). *Prescribing Outlook New Medicines - September 2013*. Retrieved from <https://www.ukmi.nhs.uk/filestore/ukmianp/PrescribingOutlook-NewMedicines2013.pdf>

UK Medicines Information. (2014). *Prescribing Outlook New Medicines - September 2014*. Retrieved from <https://www.ukmi.nhs.uk/filestore/ukmianp/2014PrescribingOutlook-NewMedicines2014-FINAL.pdf>

UK Medicines Information. (2015). *Prescribing Outlook New Medicines - September 2015*. Retrieved from <https://www.ukmi.nhs.uk/filestore/ukmianp/2015PrescribingOutlook-NewMedicines-FINAL.pdf>

UK Medicines Information. (2016). *Prescribing Outlook New Medicines - September 2016*. Retrieved from <https://www.sps.nhs.uk/wp-content/uploads/2016/09/FINAL-2016-Prescribing-Outlook-New-Medicines.pdf>

Vallejo-Torres, L., García-Lorenzo, B., Castilla, I., Valcárcel-Nazco, C., García-Pérez, L., Linertová, R., . . . Serrano-Aguilar, P. (2016). On the Estimation of the Cost-Effectiveness Threshold: Why, What, How? *Value Health, 19*(5), 558-566. doi:10.1016/j.jval.2016.02.020
